# Mothers’ and fathers’ stress and severity of depressive symptoms during the COVID-19 pandemic: actor-partner effects with parental negative emotions as a moderator

**DOI:** 10.1186/s40359-022-01016-y

**Published:** 2022-12-09

**Authors:** Rebecca Y. M. Cheung, Wing Yee Cheng, Jian-Bin Li, Eva Yi Hung Lau, Kevin Kien Hoa Chung

**Affiliations:** 1 Centre for Child and Family Science, The Education University of Hong Kong, Hong Kong, Hong Kong SAR; 2 School of Psychology and Clinical Language Sciences, University of Reading, Reading, UK; 3 Department of Early Childhood Education, The Education University of Hong Kong, Hong Kong, Hong Kong SAR

**Keywords:** Actor-partner effects, Parental stress during COVID-19, Negative emotions during COVID-19, Severity of depressive symptoms

## Abstract

**Background:**

In the face of the coronavirus disease 2019 (COVID-19) pandemic, families with young children are bombarded with new challenges and stressors. This study examined the additive and interactive effects of parental stress and negative emotions during COVID-19 on parents’ severity of depressive symptoms.

**Methods:**

Participants were 221 Chinese families involving maritally intact mothers and fathers of preschool-aged children.

**Discussion:**

Path analysis indicated that mothers’ parental stress interacted with their negative emotions, such that their stress was related to their severity of depressive symptoms only when negative emotions were high. By comparison, fathers’ stress and negative emotions were additively associated with their severity of depressive symptoms. Supporting the cumulative risk model, parental stress during COVID-19 and negative emotions were linked to parents’ severity of depressive symptoms additively or interactively, depending on the gender of the parent. These findings inform practitioners about the relevance of parents’ stress and negative emotions to their severity of depressive symptoms during the pandemic.

## Introduction

The coronavirus disease 2019 (COVID-19) pandemic not only has a long-term impact on public health, but also negatively influences children and families [1, 2]. By 13 June 2022, Hong Kong had experienced five waves of infections and accumulated 752,740 cases involving a total of 9177 deaths [3]. Although the number of cases and deaths in Hong Kong was low compared to other regions and countries worldwide, the Hong Kong government has implemented strict restrictions (e.g., closure of schools and playgrounds) to prevent the spread of the infection which is especially challenging for families with young children. The social restriction measures were particularly challenging to families in Hong Kong due to residential crowding and reliance on public space for recreation [4–6]. During the pandemic, scholars and researchers in non-medical sciences have found that stress and perceived severity of COVID-19 were detrimental to both mental and physical well-being of the general population [7–9]. Nevertheless, a topic that has received less attention is the mental health among parents during the pandemic [10, 11]. Based on the latest COVID-19 development, the pandemic is likely to last for an extensive period. Hence, the role of stress on local families should be examined to ensure appropriate support is provided to facilitate both parents' and children’s well-being [12].

### Parental stress and negative emotions: Are parents experiencing a “double whammy” of risk for depression?

Parental stress is associated with parents’ psychological distress and symptoms of depression and anxiety [13–16]. During the COVID-19 outbreak, children and parents in Hong Kong have experienced recurring and prolonged school suspension. When school is suspended, parents face competing demands because of their increased responsibilities and challenges in supervising children’s distance learning and managing their changing routines [17–19]. Due to unemployment and underemployment, a significant proportion of parents in Hong Kong and Worldwide have also experienced financial stress that has further exacerbated parents’ overall stress levels [14, 20]. Coupled with stress are negative emotions revolving around COVID-19, including fear, frustration, and loneliness in the face of an uncertain future [21, 22]. Importantly, parents who appraise COVID-19-related challenges negatively may be more prone to experiencing negative emotions [23, 24], which are linked to worse mental health and greater depressive symptoms [25–28].

**Fig. 1:**
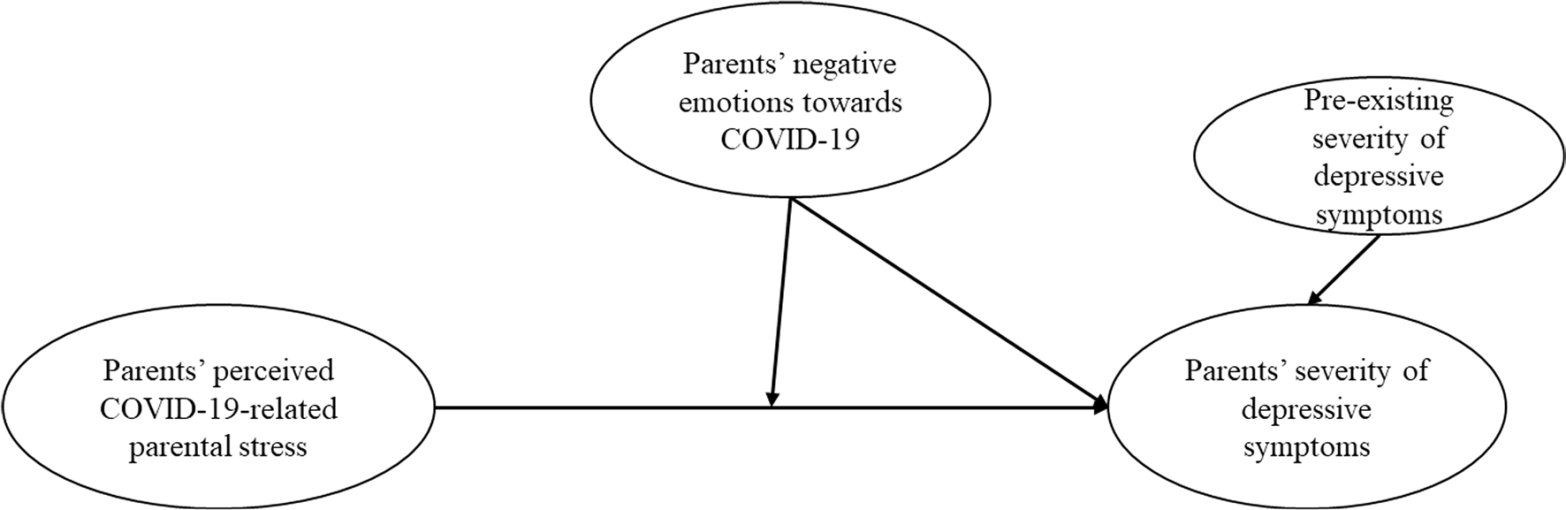
Conceptual model of parental stress and negative emotions as correlates of parents’ depressive symptoms

According to the cumulative risk model [29–31], multiple risks may be additively or interactively associated with people’s vulnerability to maladjusted outcomes. After all, it is not necessarily a specific risk but a number of risks leading to maladjustment [3, 32]. Regarding the additive effect of risks, parents’ negative emotions and parental stress during COVID-19 may be accumulatively associated with the severity of depressive symptoms. As for the interactive effects of risks, parents experiencing greater negative emotions may exacerbate the adverse effect of parental stress during COVID-19 on their severity of depressive symptoms (see Fig. 1 for conceptual model).

Despite the potential link between stress, negative emotions, and depression, few studies have investigated whether stressful parents who feel particularly negative during the pandemic may experience a “double whammy” of risk for depression (see also [33]). Given that the severity of parental depressive symptoms can significantly undermine family functioning and children’s psychosocial and behavioral adjustment [34–37], the relations between parents’ stress, emotions, and depression deserve empirical attention.

### Dyadic relations between mothers and fathers

According to the actor-partner interdependence model [38], mothers’ and fathers’ psychological functioning are dyadically related [39]. Of note, recent studies conducted in the Chinese context indicated that mothers’ dysregulation of emotions predicted not only their own emotion dysregulation a year later, but also their spouses’ and their children’s emotion dysregulation [40]. In another study, mothers’ emotion dysregulation predicted their own and their spouses’ supportive reactions (i.e., expressive encouragement, problem-focused responses, and emotion-focused responses [41]) to children’s negative emotions [39]. Fathers’ emotion dysregulation, however, only predicted their own supportive reactions to children’s negative emotions [39]. In a third study, mothers’ and fathers’ interparental conflict behavior had both actor and partner effects on their future conflict behavior and mindful parenting practices [42]. These findings illustrated dyadic relations between mothers’ and fathers’ behavior, particularly in the Chinese context.

In the midst of the COVID-19 pandemic, recent findings indicated that mothers’ and fathers’ mental health were both associated with family dynamics (e.g., parental positivity, coparenting support) [43]. During the pandemic, mothers’ and fathers’ parental stress, negative emotions, and pre-existing depression may reciprocate to affect their own and their spouses’ severity of depressive symptoms. Hence, it is crucial to elucidate potential actor and partner effects on parental depression.

### The present study

The current research examined the extent to which parental stress during COVID-19 was related to parents’ severity of depressive symptoms. Based on the cumulative risk model [29–31], we further investigated the additive and interaction effects of risks during COVID-19, including parental stress and parents’ negative emotions, on their severity of depressive symptoms. In our hypothesized model, child's age, family income, and extra help (e.g., childcare) from domestic helpers were added as covariates with paths directing to parents’ severity of depressive symptoms (see also [44]) and parenting stress (see also [45]). We hypothesized that mothers’ and fathers’ parental stress and negative emotions during COVID-19 would be additively related to their severity of depressive symptoms, over and above their pre-existing severity of depressive symptoms (H1). We also hypothesized that parental stress during COVID-19 would interact with their negative emotions, such that greater negative emotions during COVID-19 would exacerbate the negative relation between parental stress and severity of depressive symptoms, regardless of the gender of the parent (H2). We further hypothesized both actor and partner effects of mothers’ and fathers’ stress during COVID-19, negative emotions, and pre-existing severity of depressive symptoms on their severity of depressive symptoms during the pandemic (H3).

## Methods

### Participants

This study is part of a longitudinal study investigating family dynamics and well-being [35]. Data were collected pre-COVID-19 on the baseline severity of parental depressive symptoms in March–June 2019. Follow-up data on parents’ parental stress, emotions, and severity of depressive symptoms were collected at Time 2 (T2) during the pandemic in October 2020–May 2021.

Participants were 235 families from Hong Kong involving mothers and fathers of preschool-aged children. All participating parents were proficient in Chinese. For the purpose of this study, four single-parent families and 10 families with a child with special education needs were excluded from the analyses, given that their family stress and dynamics might be different from the rest of the families [46, 47]. The final sample consisted of 221 families with married couples who had participated at both time points without attrition. At baseline, the mothers were 25–46 years of age (*M* = 36.45, *SD* = 4.35) and the fathers were 25–72 years of age (*M* = 39.66, *SD* = 6.18). The children’s age range was 40.02–55.66 months (*M* = 46.44 months, *SD* = 3.60). The median monthly household income of our sample was HK$40,001–50,000 (approximately US$5135.41–6419.10), which was higher than the median monthly household income (HK$34,000) of the Hong Kong general population [48]. In this sample, 92.56% of fathers and 47.69% of mothers were employed full-time and 39.52% of families hired domestic helpers.

Table 1 shows the demographic information of the current sample, including age and household income per month. Table 2 shows the percentages of parents who reported that they knew someone who had contracted or passed away due to COVID-19.

**Table 1:** Demographic information of the final sample (N = 221)

Variable	*M* (*SD*)/%
Families involving married couples	97.74%
Percentage of fathers in the sample	97.74%
Percentage of mothers in the sample	100.00%
Mothers’ age (in years, at baseline)	36.45 (4.35)
Fathers’ age (in years, at baseline)	39.66 (6.18)
Child age (in years, at baseline)	3.87 (.30)
Household income per month	
1. < HK$10,000 (< US$1282)	1.86%
2. HK$10,001–15,000 (US$1282–1923)	6.51%
3. HK$15,001–20,000 (US$1923–2564)	11.63%
4. HK$20,001–50,000 (US$2564–6410)	44.64%
5. HK$50,001–80,000 (US$6410–10,256)	15.81%
6. HK$80,001–100,000 (US$10,256–12,820)	4.65%
7. > HK$100,000 (> US$12,820)	14.88%
Employed full-time	
Mothers	47.69%
Fathers	92.56%
Families which had domestic helpers	39.52%

**Table 2:** Percentages of parents who knew someone contracted or passed away due to COVID-19

	Knowing someone who contracted COVID-19		Knowing someone who passed away due to COVID-19	
	Father	Mother	Father	Mother
Partner/Spouse	0% (*n* = 0)	0% (*n* = 0)	0% (*n* = 0)	0% (*n* = 0)
Child	0% (*n* = 0)	0% (*n* = 0)	0% (*n* = 0)	0% (*n* = 0)
Family member	0% (*n* = 0)	0.90% (*n* = 2)	0.45% (*n* = 1)	0.90% (*n* = 2)
Friend	2.71% (*n* = 6)	2.26 (*n* = 5)	0.45% (*n* = 1)	0% (*n* = 0)
Someone else they know	13.57% (*n* = 30)	7.69 (*n* = 17)	1.36% (*n* = 3)	1.36% (*n* = 3)

### Procedures

The study was approved by the Human Research Ethics Committee at The Education University of Hong Kong (Reference number: 2018-2019-0037) and met the ethical standards of the Declaration of Helsinki and the American Psychological Association. Throughout the study, all recruitment and communication with families were done through five kindergartens in Hong Kong. Written informed consent was obtained from parents prior to the beginning of the study. Mothers and fathers received their own packets of questionnaires through the kindergarten and were asked to complete the questionnaires independently at home. At both baseline and T2, the packet distribution procedures were the same and parents completed the questionnaires on paper for approximately 30 to 45 min. At the end of the study, each parent was compensated with a HK$50 supermarket coupon (~ US$6.42) for their time and effort.

### Measures

#### Parental stress during COVID-19

At T2, an adapted checklist developed by Brown and colleagues [10] was used to assess mothers’ and fathers’ perceived parental stress during COVID-19. The checklist included three items and parents responded to the question, “Have you ever experienced the following areas of parental stressors during the outbreak of COVID-19: (1) parent’s relationship/interactions with child(ren), (2) child(ren)’s physical health, and (3) child(ren)’s academic/learning?”. The scale ranged from 0 (*no*) to 1 (*yes*). The scores were summed, with higher scores indicating greater parental stress during COVID-19. The measure was translated from English to Chinese by trained research assistants following the back-translation procedures [49]. The Cronbach’s alphas of mothers’ and fathers’ reports were 0.64 and 0.76, respectively.

#### Parents’ negative emotions during COVID-19

At T2, mothers and fathers recalled their experiences of negative emotions following the outbreak of COVID-19. The 8-item Negative Emotions subscale was administered in Chinese and was rated on a 5-point scale [24]. The scale of negative emotions ranged from 1 (*much lower than the days before the outbreak*) to 5 (*much greater than the days before the outbreak*). Sample items included “feel worried”, “feel lonely”, “feel nervous”, “feel angry”, and “feel anxious”. The scores were summed, with higher scores indicating participants’ greater levels of negative emotions following COVID-19. The Cronbach’s alphas of mothers’ and fathers’ reports were 0.96 and 0.97, respectively.

#### Parents’ severity of depressive symptoms

At baseline and T2, the Chinese version of the Patient Health Questionnaire [50] was used to assess mothers’ and fathers’ severity of depressive symptoms over time. The 9-item scale ranged from 0 (*none*) to 3 (*almost every day*). Sample items included, “little interest or pleasure in doing things”, “feeling down, depressed, or hopeless”, “trouble concentrating on things, such as reading the newspaper or watching television”, and “feeling tired or having little energy”. The scores were summed to a score ranging from 0 to 27, with higher scores indicating greater severity of depressive symptoms. According to previous research [51–53], the PHQ-9 demonstrated adequate criterion validity, good test–retest reliability, and good internal consistency. The Cronbach’s alphas of mothers’ and fathers’ reports were 0.90 and 0.82 at baseline, respectively, and 0.90 and 0.88 at T2, respectively.

### Statistical analyses

A path model was conducted via MPLUS, Version 8.3 [54] to examine the additive and interaction effects of parents’ perceived parental stress during COVID-19 and negative emotions during COVID-19 on their severity of depressive symptoms, over and above the effects of household income, additional help from domestic helpers, child’s age, and parents’ pre-existing severity of depressive symptoms. To facilitate interpretability, parents’ perceived parental stress during COVID-19 and negative emotions during COVID-19 were centered on the mean. To examine the potential actor-partner interdependent effects [38], mothers’ and fathers’ perceived parental stress during COVID-19, negative emotions, and pre-existing severity of depressive symptoms were entered to the same path model as correlates of severity of depressive symptoms during the pandemic. Missing data at the item or subscale level were handled by the full information maximum likelihood method. The fit of the model was considered as acceptable if the CFI value was 0.90 or above [55], the RMSEA value was 0.10 or below [56], and the SRMR value was 0.05 or below [57].

## Results

Table 3 shows the means, standard deviations, and correlations among the variables under study. Paired-sample t-tests showed that mothers did not differ from fathers on their levels of parental stress during COVID-19, negative emotions during COVID-19, and severity of depressive symptoms (*p*s > 0.05). There was no significant mean differences between the severity of depressive symptoms at baseline and T2 for mothers, *t*(215) = 0.41, *p* = .69, *M*_*Time 1*_ = 3.49, *SD* = 4.01, *M*_*Time 2*_ = 3.40, *SD* = 4.25, and for fathers, *t*(211) = -0.91, *p* = .37, *M*_*Time1*_ = 3.26, *SD* = 3.48, *M*_*Time2*_ = 3.51, *SD* = 4.02. Mothers’ and fathers’ stress, emotions, and severity of depressive symptoms were correlated with each other, *p*s < 0.05.

**Table 3:** Means, standard deviations, and correlations among all variables

Variable	(1)	(2)	(3)	(4)	(5)	(6)	(7)	(8)	(9)	(10)	(11)
(1) Household income	–										
(2) Additional help from domestic helpers (0 = no; 1 = yes)	.59***	–									
(3) Child’s age at Time 2 (months)	.07	.07	–								
(4) Mothers’ COVID-19-related parental stress	− .07	− .10	− .04	–							
(5) Fathers’ COVID-19-related parental stress	− .09	.01	− .07	.40***	–						
(6) Mothers’ negative emotions during COVID-19	.14*	.02	.04	.23***	.21**	–					
(7) Fathers’ negative emotions during COVID-19	.13	.03	.07	.23***	.25***	.41***	–				
(8) Mothers’ severity of depressive symptoms	− .13	− .15*	− .12	.23***	.14*	.37***	.19**	–			
(9) Fathers’ severity of depressive symptoms	− .10	− .03	− .06	.22***	.36***	.18**	.26***	.42***	–		
(10) Pre-existing severity of mothers’ depressive symptoms	− .03	.08	.14*	.22***	.17*	.30***	.15*	.67***	.31***	–	
(11) Pre-existing severity of fathers’ depressive symptoms	− .03	.03	.00	.20**	.28***	.15*	.14*	.24***	.42***	.32***	–
*M*	6.17	.40	70.48	1.24	1.00	3.18	3.12	3.39	3.51	3.52	3.32
*SD*	3.27	.49	3.62	1.06	1.12	.82	.83	4.24	3.94	3.99	3.57

*p = */* < .05, **p = */* < .01, ***p = */* < .001. Monthly household income scale ranging from 0 to 12: 0 = less than HK$5000; 1 = HK$5001–10,000; 2 = HK$10,001–15,000; 3 = HK$15,001–20,000; 4 = HK$20,001–30,000; 5 = HK$30,001–40,000; 6 = HK$40,001–50,000; 7 = HK$50,001–60,000; 8 = HK$60,001–70,000; 9 = HK$70,001–80,000; 10 = HK$80,001–90,000; 11 = HK$90,001–100,000; 12 = over HK$100,001

**Fig. 2:**
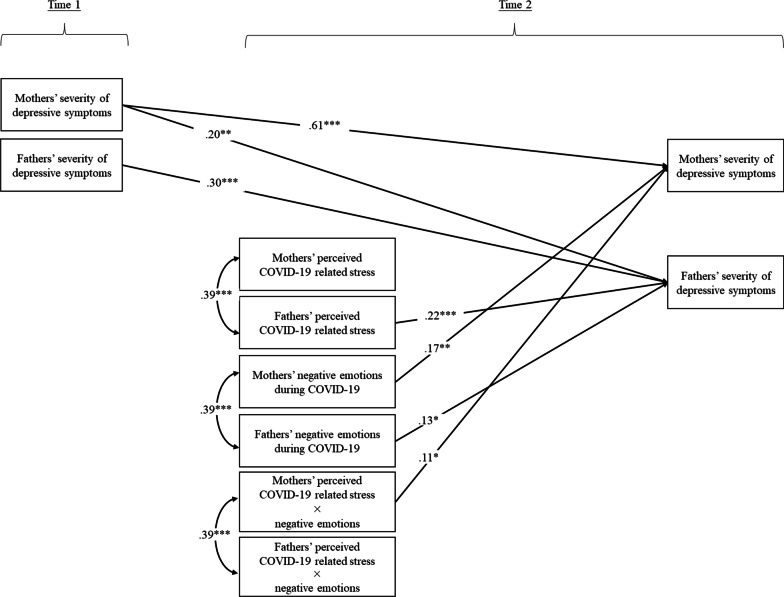
Parental stress, negative emotions, and pre-existing depressive symptoms as correlates of parents’ depressive symptoms during the pandemic. *Note:* χ^2^(4) = 9.42, *p* > .05, CFI = .98, RMSEA = .08, SRMR = .03. Family income, help from domestic helpers, and child's age were included as covariates of parents’ severity of depressive symptoms. Non-significant paths are not depicted in the figure for clarity. *p = / < .05, **p = / < .01, ***p = / < .001

The path model fit adequately to the data, *χ*^2^(4) = 9.42, *p* > .05, CFI = 0.98, RMSEA = 0.08, SRMR = 0.03 (see Fig. 2 and Table 4 for details). Specifically, mothers’ perceived parental stress during COVID-19 was correlated with fathers’ perceived parental stress during COVID-19 (*r* = .39, *p* < .001). Mothers’ negative emotions during COVID-19 were also correlated with fathers’ negative emotions during COVID-19 (*r* = .39, *p* < .001). The interaction terms were also correlated between mothers and fathers (*r* = .39, *p* < .001).

**Table 4:** Parameter estimates of the model

Parameter	Unstandardized *B* (*SE*)	Standardized β
*Structural model*		
Mothers’ COVID-19-related parental stress		
$\to$Mothers’ severity of depressive symptoms	.22 (.21)	.06
$\to$Fathers’ severity of depressive symptoms	− .13 (.25)	− .04
Fathers’ COVID-19-related parental stress		
$\to$Mothers’ severity of depressive symptoms	− .17 (.21)	− .04
$\to$Fathers’ severity of depressive symptoms	.79 (.23)	.22***
Mothers’ negative emotions during COVID-19		
$\to$Mothers’ severity of depressive symptoms	.89 (.29)	.17**
$\to$Fathers’ severity of depressive symptoms	− .05 (.33)	− .01
Fathers’ negative emotions during COVID-19		
$\to$Mothers’ severity of depressive symptoms	.07 (.28)	.02
$\to$Fathers’ severity of depressive symptoms	.63 (.32)	.13*
Mothers’ COVID-19-related parental stress × negative emotions		
$\to$Mothers’ severity of depressive symptoms	.51 (.25)	.11*
$\to$Fathers’ severity of depressive symptoms	− .33 (.29)	− .08
Fathers’ COVID-19-related parental stress × negative emotions		
$\to$Mothers’ severity of depressive symptoms	.03 (.22)	.01
$\to$Fathers’ severity of depressive symptoms	.35 (.15)	.09
*Control variables*		
Pre-existing severity of mothers’ depressive symptoms		
$\to$Mothers’ severity of depressive symptoms	.64 (.06)	.61***
$\to$Fathers’ severity of depressive symptoms	.20 (.07)	.20**
Pre-existing severity of fathers’ depressive symptoms		
$\to$Mothers’ severity of depressive symptoms	.04 (.06)	.03
$\to$Fathers’ severity of depressive symptoms	.33 (.07)	.30***
Household income		
$\to$Mothers’ severity of depressive symptoms	.02 (.08)	.02
$\to$Fathers’ severity of depressive symptoms	.03 (.10)	.02
Additional help from domestic helpers		
$\to$Mothers’ severity of depressive symptoms	− 1.71 (.53)	− .20
$\to$Fathers’ severity of depressive symptoms	− .56 (.60)	− .07
Child’s age at Time 2		
$\to$Mothers’ severity of depressive symptoms	− .04 (.06)	− .04
$\to$Fathers’ severity of depressive symptoms	− .02 (.07)	− .02
*Covariance*		
Mothers’ COVID-19-related parental stress		
$\leftarrow \to$Fathers’ COVID-19-related parental stress	.46 (.08)	.39***
Mothers’ negative emotions during COVID-19		
$\leftarrow \to$Fathers’ negative emotions during COVID-19	.27 (.05)	.39***
Mothers’ COVID-19-related parental stress × negative emotions		
$\leftarrow \to$Fathers’ COVID-19-related parental stress × negative emotions	.34 (.07)	.39***
Household income		
$\leftarrow \to$Additional help from domestic helpers	.92 (.13)	.59***
*Error covariance*		
Mothers’ severity of depressive symptoms		
$\leftarrow \to$Fathers’ severity of depressive symptoms	2.86 (.68)	.30***

*p < .05, **p < .01, ***p < .001

**Fig. 3:**
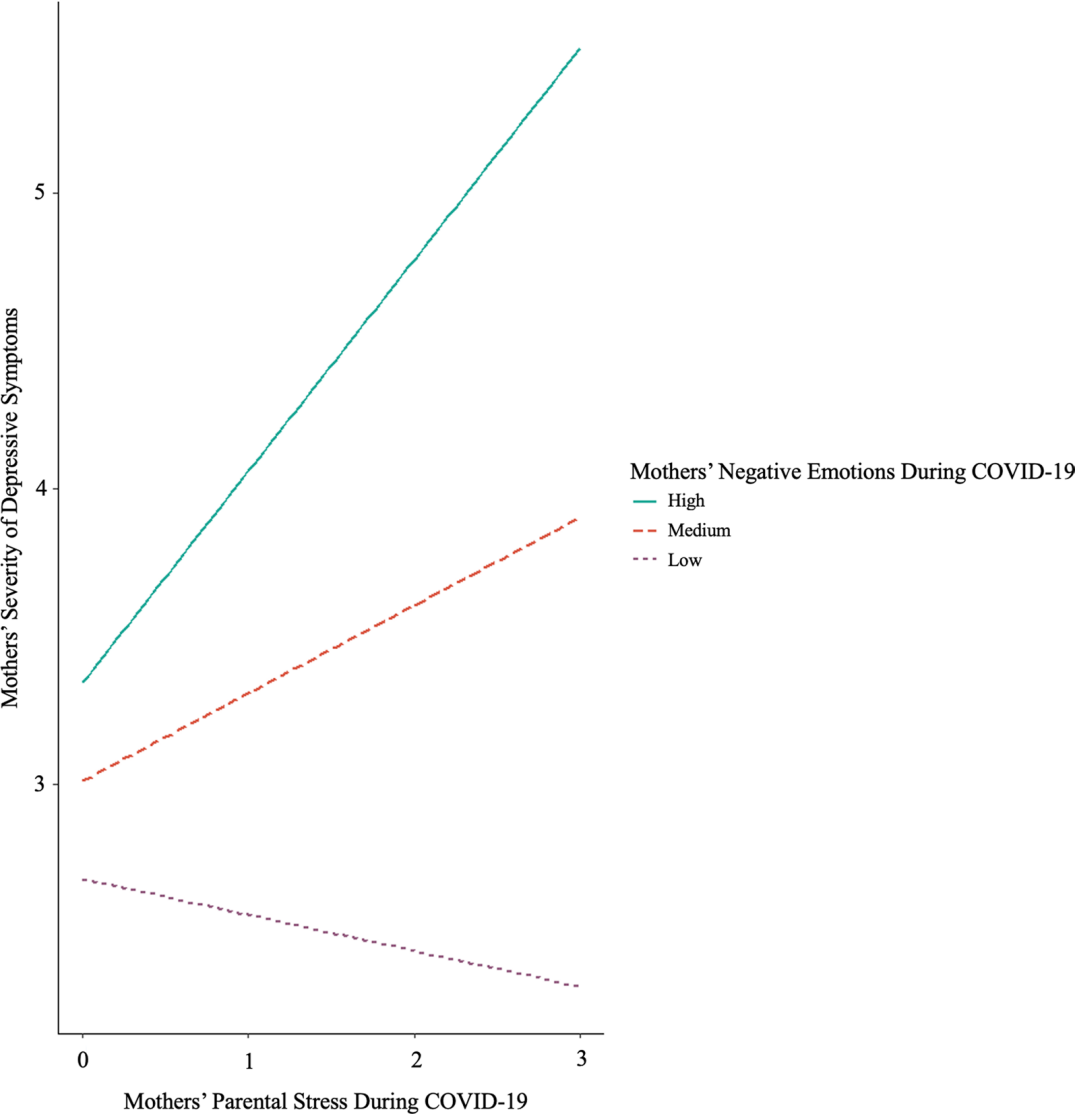
Interaction effects of mothers’ stress and negative emotions on the severity of depressive symptoms

After controlling for the effects of household income, additional help from domestic helpers, child age at Time 2, and pre-existing severity of parental depressive symptoms, fathers’ perceived parental stress during COVID-19 and negative emotions during COVID-19 were associated with fathers’ severity of depressive symptoms (β = 0.22, *p* = .001; β = 0.13, *p* = .047, respectively). However, the interaction between fathers’ perceived parental stress during COVID-19 and negative emotions during COVID-19 was not related to fathers’ severity of depressive symptoms (*p* = .162). Mothers’ perceived parental stress during COVID-19 was not related to mothers’ severity of depressive symptoms (*p* = .297). However, their negative emotions during COVID-19 were associated with mothers’ severity of depressive symptoms (β = 0.17, *p* = .002). Moreover, mothers’ perceived parental stress during COVID-19 and negative emotions during COVID-19 were interactively associated with mothers’ severity of depressive symptoms (β = 0.11, *p* = .039). According to the post-hoc simple slopes test, the relation between mothers’ perceived parental stress during COVID-19 and mothers’ severity of depressive symptoms was significantly positive when mothers’ negative emotions during COVID-19 was high (i.e., 1 *SD* above the mean), β = 0.30, *p* < .001, but not significant when it was low (i.e., 1 *SD* below the mean), *p* = .138 (see Fig. 3 for details).

Despite the significant correlations of the exogenous variables between mothers and fathers (*p*s < 0.001, see Table 3 for details), no partner effects were found in the path model between mothers’ and fathers’ stress and negative emotions as correlates of severity of depressive symptoms (*p*s > 0.05). However, mothers’ pre-existing severity of depressive symptoms was associated with fathers’ subsequent severity of depressive symptoms (β = 0.20, *p* = .005) as well as their own subsequent severity of depressive symptoms (β = 0.61, *p* < .001).

### Supplementary analysis

Based on the actor-partner interdependence model [38], two additional interaction terms between mothers’ versus fathers’ own negative emotions and their partners’ parental stress during COVID-19 were included in the model. However, the path model fit poorly to the data, *χ*^2^(30) = 82.46, *p* < .001, CFI = 0.75, RMSEA = 0.10, SRMR = 0.07. Hence, the present findings did not support the addition of dyadic interaction effects on the severity of depressive symptoms.

## Discussion

The present study revealed unique associations between mothers’ and fathers’ stress, negative emotions, and the severity of depressive symptoms during the COVID-19 pandemic. Supporting the literature [58, 59], both mothers’ and fathers’ negative emotions were associated with their respective severity of depressive symptoms. In addition, fathers’ parental stress during COVID-19 was related to their own severity of depressive symptoms. Contrary to the additive effects of fathers’ stress and emotions on depression, mothers’ parental stress interacted with their negative emotions, such that maternal stress was related to mothers’ severity of depressive symptoms only when their negative emotions during COVID-19 were high. Supporting the cumulative risk model [30, 60], parental stress and negative emotions during COVID-19 were both linked to the severity of parental depressive symptoms additively or interactively, depending on the gender of the parent.

In the face of an uncertain future, parents are likely to experience negative emotions such as fear, frustration, and loneliness during the pandemic [21, 22]. Situated in the context of Hong Kong, whereby residential crowding is common [4], parents may be more likely to experience a greater level of stress and conflict as a result of social restrictions, school closure, and work-from-home policy [61, 62]. Interestingly, differential findings emerged as a function of parents’ gender: Fathers’ parental stress and negative emotions during COVID-19 were additively related to their own severity of depressive symptoms, whereas mothers’ stress and negative emotions were interactively related to their own severity of depressive symptoms. That is, fathers’ parental stress during COVID-19 was linked to their worse severity of depressive symptoms, regardless of their level of negative emotions, whereas mothers’ stress was linked to their severity of depressive symptoms only when they experienced a high level of negative emotions. The findings may be explained by gender role expectations in the traditional Chinese culture [63, 64]. Compared to fathers, mothers generally take the nurturing role of caregiving and are more involved in children’s day-to-day activities. On the contrary, fathers are likely to preserve authority by assuming the role of a provider and a disciplinarian. Even though coparenting practices have become increasingly common in contemporary Chinese societies [65], gender role expectations remain culturally prevalent [66]. In this sample, 92.56% of fathers and 47.69% of mothers did report that they were employed full-time, implying that mothers were more likely to have heavier caregiving responsibilities and might have experienced some parental stress pre-COVID-19. As mothers were likely to have spent a significant amount of time with their children, the additional parental stress during COVID-19 alone did not appear to be pivotal to their mental well-being, unless they felt particularly negative about COVID-19. As such, an interaction effect of parental stress during COVID-19 and negative emotions on the severity of depressive symptoms emerged for mothers. On the contrary, for fathers, both the parental stress during COVID-19 and negative emotions precipitated their severity of depressive symptoms, potentially because they were not accustomed to spending most of the time at home and being highly involved in children’s daily activities. Even though both parents experience a “double whammy” of risk for elevated severity of depressive symptoms, thereby supporting the cumulative risk model [30, 60], our study indicated differential findings as a function of the parents’ gender. Furthermore, when additional interaction terms between mothers’ versus fathers’ own negative emotions and their partners’ parental stress during COVID-19 were included, the model fit was poor. Future studies should explore the dyadic interaction effects of stress and emotions on parents’ severity of depressive symptoms in a larger sample.

### Limitations and future directions

The present findings must be interpreted in light of several limitations. First of all, given that we only had a single time point of data regarding parents’ experiences of stress and negative emotions, we were unable to test the directionality of effects between parents’ stress, emotions, and severity of depressive symptoms. Notably, some studies showed that family stress and parental depressive symptoms bidirectionally predicted one another over time [16], whereas others indicated unidirectional effects of parents’ depressive symptoms on parenting stress [67] or unidirectional effects of parental stress on parents’ depressive symptoms [15]. Although the present study included the baseline severity of depressive symptoms as control variables, which informed the field on how parents' depressive symptoms were shaped by the pandemic [68], future studies should further clarify the directionality of effects by collecting longitudinal data to examine parents’ experiences of stress, emotions, and depressive symptoms in cross-lagged panel models. Next, according to family systems theory [69, 70], interparental and parent–child dynamics are closely connected [71, 72]. Therefore, future studies should examine not only the relations between mothers and fathers, but also parent and child effects on different family members’ mental health. Third, as discussed earlier, 92.56% of fathers and 47.69% of mothers in this sample were employed full-time. The difference between the percentages might have implied mothers’ and fathers’ differential parent–child interactions and time spent with children. Future research should take account of parents’ employment status and further examine gender role and family role in relation to parental stress and mental health. Fourth, mothers’ report of parental stress during COVID-19 had a relatively low internal consistency at Cronbach’s alpha = 0.64. Hence, the findings should be interpreted with caution. Fifth, despite the stress revolving around the pandemic (e.g., school closure, job loss), the number of COVID-19-infected individuals in Hong Kong was very low at the time of data collection in October 2020–May 2021. A closer examination of the data reflected a floor effect showing a low percentage of parents who knew someone contracted or passed away due to COVID-19 (see Table 2). As the variables were highly skewed, we decided not to include them as covariates. However, knowing a family member or a friend who suffered from COVID-19 might have added substantial stress for the parents. Hence, future studies should control for its potential effects on parental stress [73]. Sixth, even though we asked the parents about their employment status, we did not have specific information on whether they were full-time and essential workers or how much quality time they spent with their children per week, which might have been covariates of parental stress. Similarly, we did not examine the number of children in a household as a family covariate of parental stress or severity of depressive symptoms during the pandemic. Future research should take these variables into account and include other family covariates, such as family conflict, coparenting support, and mindful parenting, as they may also be linked to parents’ and children’s adjustment during the pandemic [73–76]. Seventh, we did not measure general parenting stress in this study. As such, we were unable to conclude whether parental stress during COVID-19 was related to parents’ severity of depressive symptoms over and above general parenting stress. Finally, the present study only utilized self-report. To increase the objectivity of the findings, future studies should include other assessments such as partner-report, observational measures, as well as biophysiological measures of stress.

## Conclusions

The present study lends support to the cumulative risk model [30, 60] and advances the literature for the effects of parental stress and negative emotions on parents’ severity of depressive symptoms during COVID-19. The study also indicated differential findings as a function of parents’ gender. Specifically, both mothers’ and fathers’ negative emotions were associated with their respective severity of depressive symptoms. Fathers’ parental stress during COVID-19 was related to their own severity of depressive symptoms, whereas mothers’ parental stress interacted with their negative emotions, such that maternal stress was related to their severity of depressive symptoms only when their negative emotions during COVID-19 were high. A key take-home message is that parental stress and negative emotions were additively or interactively associated with the severity of parental depressive symptoms during the pandemic, depending on the gender of the parent. Psychological interventions geared toward stress reduction and mental health promotion during the pandemic merit future investigation.

## Data Availability

The datasets generated and/or analysed during the current study are not publicly available due to confidentiality and ethical reasons but are available from the corresponding authors upon reasonable request.
